# Hughes–Stovin Syndrome: A Rare Cause of Pulmonary Artery Aneurysm and Thrombosis—A Case Report

**DOI:** 10.1111/1756-185X.70227

**Published:** 2025-04-14

**Authors:** Abdul Qadir, Amal Wael Abdellatif, Aamir Waheed, Mamunul Islam

**Affiliations:** ^1^ Department of Medicine Hamad Medical Corporation HGH Doha Qatar; ^2^ Medical Education Department Hamad Medical Corporation Doha Qatar

## Abstract

Hughes–Stovin Syndrome (HSS) is a rare, life‐threatening condition characterized by thrombophlebitis and pulmonary artery aneurysms, often viewed as a variant of Behçet's disease. Its diagnosis and management are challenging due to its rarity and overlapping symptoms with other vascular disorders. A 33‐year‐old male presented with massive hemoptysis and a history of recurrent oral and scrotal ulcers. Imaging revealed bilateral pulmonary emboli, a pulmonary artery aneurysm, and a large right atrial thrombus. These findings, coupled with his clinical history, led to a diagnosis of HSS. A multidisciplinary team initiated anticoagulation for the thrombus and immunosuppressive therapy (steroids, cyclophosphamide). Follow‐up imaging showed improvement in both the aneurysm and thrombus. This case highlights the complexity of diagnosing and managing HSS. Early recognition, supported by a collaborative approach, is critical to improving outcomes in this rare syndrome.

1

Hughes–Stovin Syndrome (HSS) is a very rare and complex clinical disorder, primarily characterized by thrombophlebitis and the development of multiple pulmonary and/or bronchial artery aneurysms [1]. The syndrome is often considered a variant of Behçet's Disease (BD) due to overlapping clinical features, particularly its vascular manifestations [1, 2, 3]. However, HSS presents unique challenges, especially in terms of diagnosis and treatment, owing to its rarity—around 57 cases have been reported in the English medical literature [4].

Patients mostly men aged 12–40 typically present with symptoms such as cough, dyspnea, fever, chest pain, and hemoptysis [5]. These symptoms are associated with massive fatal hemoptysis due to the presence of pulmonary artery aneurysms, which are prone to rupture, causing death [3]. Its etiology remains poorly understood, with hypotheses suggesting infectious or vascular causes, though no definitive pathogen has been identified [1]. Given its severe vascular nature, HSS is often life‐threatening if not promptly treated.

This case report aims to shed light on a patient with HSS, focusing on the diagnostic challenges and treatment considerations in managing arterial, venous, and intracardiac manifestations. Early recognition and a multidisciplinary approach are essential for improving patient outcomes in such complex cases.

2

A 33‐year‐old male visited our emergency facility with the complaint of a productive cough and blood in sputum. The blood in sputum was initially scanty in quantity for the past 1 month but turned out to be massive a few days before admission. He also experienced occasional pain on both sides of his chest, particularly associated with coughing. There was no history of fever, sore throat, breathlessness, night sweats, and wheezing. He did not report any weight loss or loss of appetite. He did not have a history of recent travel or sick contact. He does not smoke or drink alcohol.

His past medical history was significant for approximately 20 recurrent episodes of oral ulcerations over the last 2 years. He experienced a painful scrotal ulceration 1 year back. He had no other significant medical history. He was vitally stable. On physical examination, the patient was pale. A superficial ulcer was observed on the medial side of the first metatarsophalangeal joint of his right foot, which the patient attributed to trauma. The systemic examination was unremarkable. The laboratory investigations were carried out as mentioned in Table 1, demonstrating anemia, an abnormal coagulation profile, and elevated inflammatory markers.

**TABLE 1:** Labs.

Parameters	Values	References
RBC	4.5	4.5–5.5 × 10^6^/μL
WBC	7.4	4.0–10.0 × 10^4^/μL
Hgb	10.0	13.0–17.0 g/dL
Hct	32.7	40.0%–50.0%
RDW‐CV	20.8	11.6%–14.0%
MCV	73.3	83–101 fL
MCHC	30.6	31.5–34.5 g/dL
MCH	22.4	27.0–32.0 pg
PDW	11.5	9.4–10.6 fL
Platelets	282	150–410 × 10^3^/μL
MPV	9.3	9.7–11.9 fL
ESR	87	2–28 mm/h
Prothrombin time	19.7	9.4–12.5 s
INR	1.8	
APTT	34.8	25.1–36.5 s
CRP	84.5	0.0–5.0 mg/L

Abbreviations: APTT, activated partial thromboplastin time; CRP, C‐reactive protein; ESR, erythrocyte sedimentation rate; Hct, hemoglobin Hgb; hematocrit; INR, international normalized ratio; MCH, mean corpuscular hemoglobin; MCHC, mean corpuscular hemoglobin concentration; MCV, mean corpuscular volume; MPV, mean platelet volume; PDW, platelet distribution width; RBC, red blood cells; RDW‐CV, red cell distribution width coefficient of variation; WBC, white blood cells.

The radiological imaging with a chest X‐ray showed a nodular opacity in the left lower zone. To investigate further, a CT pulmonary angiogram (CTPA) was performed, which demonstrated bilateral filling defects in the pulmonary artery branches (Figure 1A,B) and a small aneurysm of the subsegmental branch of the inferior lingular branch of the left pulmonary artery (Figure 2A,B). Parenchymal changes were suggestive of alveolar hemorrhage (Figure 3A,B), and a patchy consolidation was present on the left.

**FIGURE 1:**
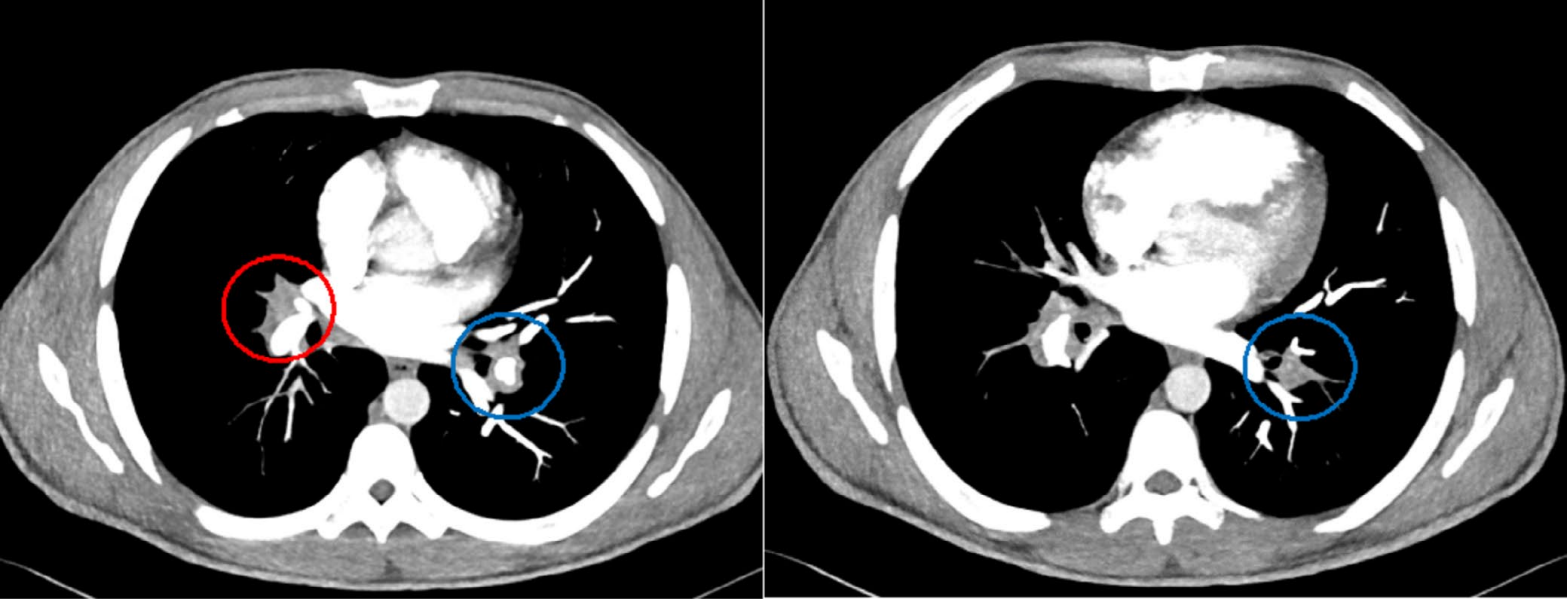
(A and B) Bilateral Pulmonary embolism. Red: right middle pulmonary artery showing filling defect. Blue: left lower basal segmental artery showing filling defect.

**FIGURE 2:**
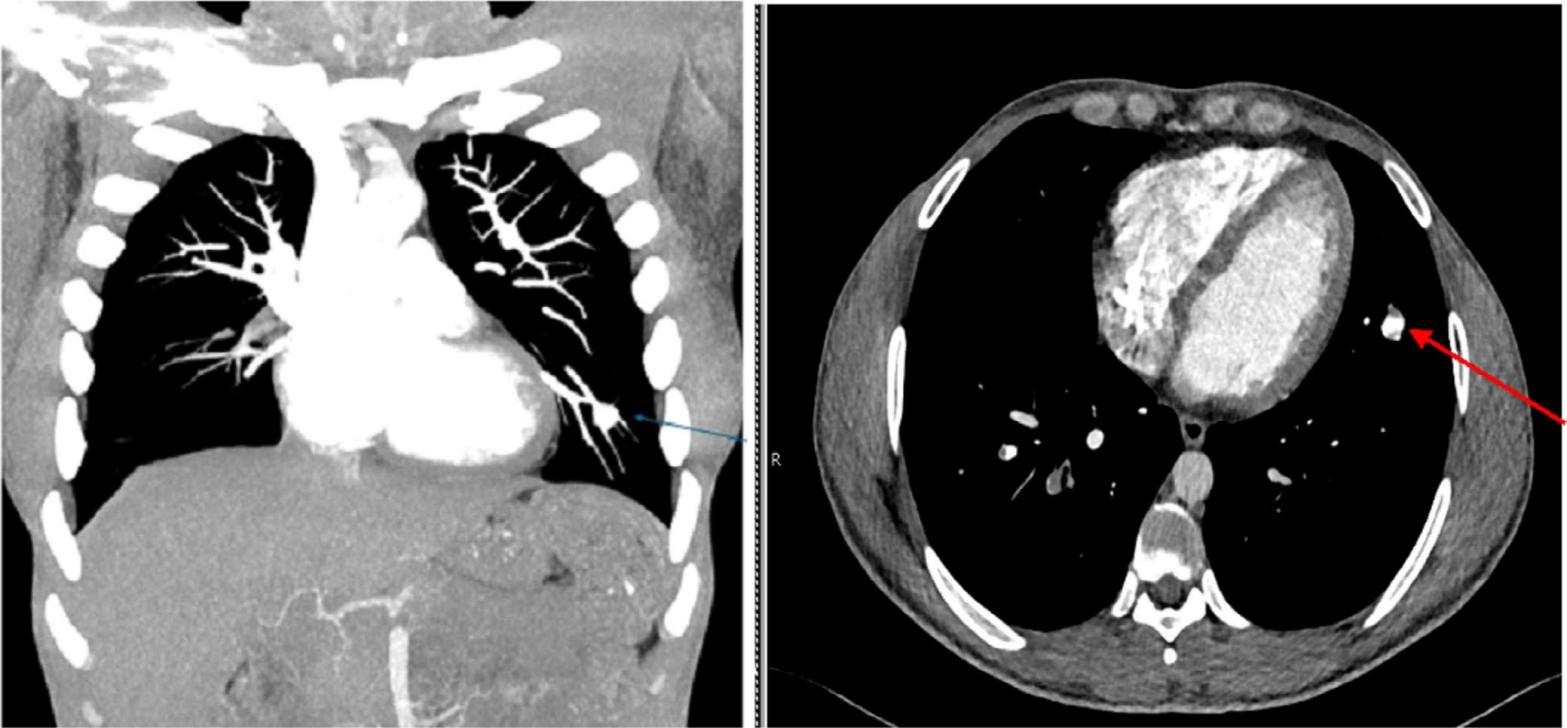
(A and B) Aneurysmal dilatation on the left is noted in a subsegmental branch of the inferior lingular artery measuring 11 mm.

**FIGURE 3:**
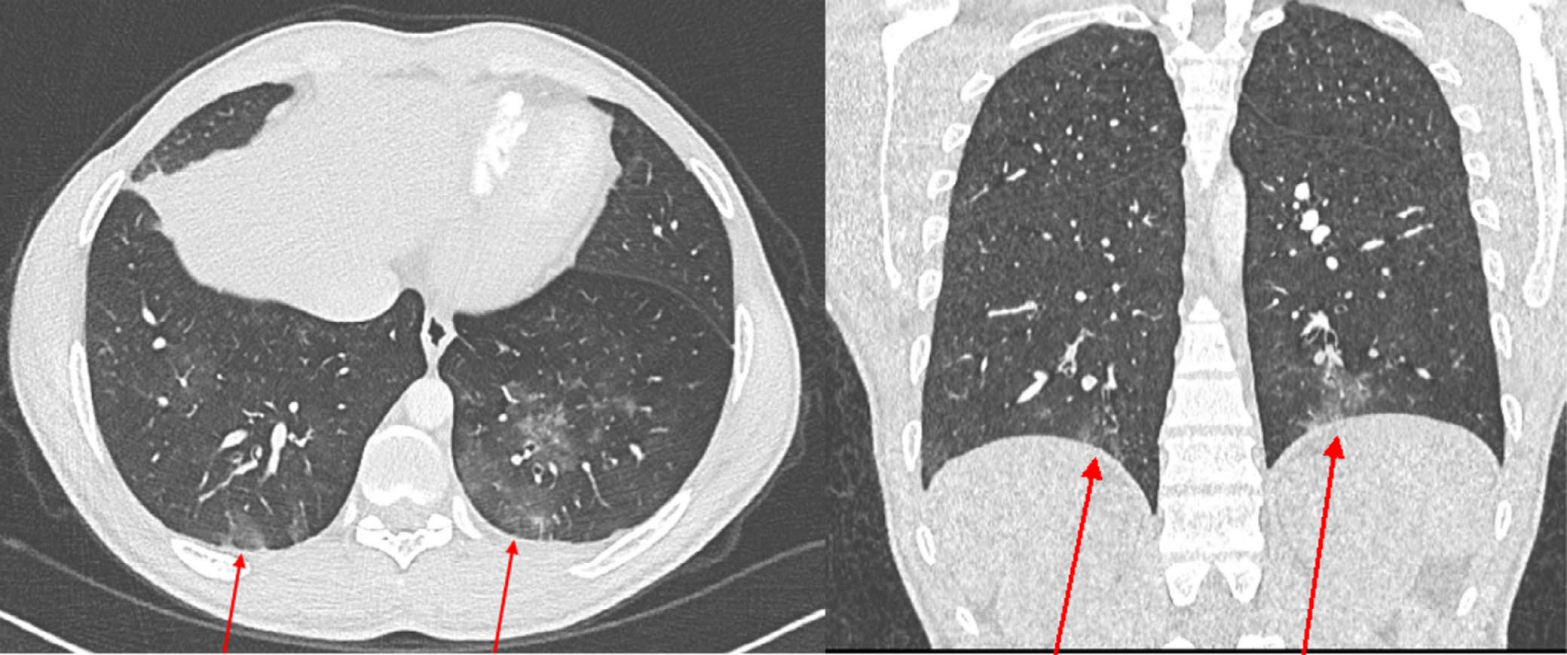
(A and B) Bilateral lower lobe alveolar hemorrhages.

A bilateral lower limb venous Doppler study revealed thrombosis in the left greater saphenous vein. Furthermore, a transesophageal echocardiogram demonstrated a large, pedunculated, highly mobile mass attached to the crista terminalis, extending into the right atrium, measuring 4.2 × 2 cm. A smaller mobile mass was attached to the tip of the larger mass, protruding through the tricuspid valve. Two small masses were also noted in the right atrial appendage. The mass did not enhance with contrast, suggesting the likely diagnosis of thrombus, though tumor or vegetations could not be completely ruled out; hence, MRI was performed.

A Cardiac MRI confirmed the presence of thrombus as a large multilobulated mass occupying the right atrial cavity along the anterolateral wall. Perfusion studies showed the mass remained predominantly hypointense with peripheral hyperintensity, suggesting thrombus formation, with blood pooling at the periphery, excluding the possibility of a tumor like myxoma or infective endocarditis vegetation. Right ventricular and left ventricular function were normal with an ejection fraction of 54%.

The cultures for infectious diseases, Hepatitis serology, and HIV serology were negative. The autoimmune screening, particularly for HLA‐B51, yielded a negative result. The coagulation study for Factor II, V, and X was normal except for a deficiency of Factor VII.

Based on the clinical presentation, imaging, and findings, the patient was diagnosed with Hughes–Stovin syndrome (HSS), a variant of Behcet's disease (BD). This was evidenced by his history of recurrent oral ulcerations, one episode of painful scrotal ulceration, the presence of a pulmonary artery aneurysm, superficial vein thrombosis (left greater saphenous vein), and the presence of intracardiac thrombi.

A multidisciplinary approach was employed, with a cardiologist, pulmonologist, hematologist, and rheumatologist collaboratively developing a management plan due to the patient's high‐risk status. Ocular screening results were negative. The multidisciplinary team decided to initiate high‐dose immunosuppressive therapy along with bone‐protective supportive agents. Intravenous (IV) methylprednisolone therapy was started on Day 3 of admission, followed by IV cyclophosphamide therapy from Day 8. Inflammatory markers showed a remarkable decrease, with C‐reactive protein (CRP) levels changing as follows: 84 → 112.6 → 66.3 → 35.5 → 21.4 → 13.2 → 9.9. The cardiologist initiated anticoagulation therapy for the right atrial (RA) thrombus under close monitoring. A follow‐up computed tomography pulmonary angiography (CTPA) performed in the third week of admission showed a 6 mm reduction in aneurysm size (from 11 to 6 mm), indicating an early response to treatment.

3

This case report highlights several important diagnostic and management considerations for the clinically heterogeneous syndrome that is Hughes–Stovin Syndrome (HSS). Our patient, who has already been suffering from multiple mild sequelae of undiagnosed HSS prior to his presentation, was brought to medical attention because of hemoptysis, leading to the diagnosis of very distinct vascular manifestations of the disease.

HSS carries significant morbidity and mortality, in addition to considerable social and economic burdens [6]. All vascular complications of HSS, including lower limb deep venous thrombosis and the less common but more dreaded complications like pulmonary artery involvement (PAI) and intracardiac thrombi, tend to preferentially happen in the male population, in whom they run a very severe course [6]. Interestingly, HSS has “cross‐associations” such that between intracardiac thrombi and PAI, as well as between intracardiac thrombi, Budd‐chiari syndrome, and Superior vena cava (SVC) syndrome. Clinicians should screen patients for multi‐territorial vascular involvement when dealing with HSS, as it often affects multiple vascular territories. The thrombosis in this condition is driven by inflammation, where cytokines activate neutrophils, leading to altered fibrinogen that resists breakdown [7]. This explains why immunosuppression is the primary treatment to prevent recurrence, as supported by clinical trials.

Our patient's tetrad includes characteristic vascular findings of deep venous thrombosis (DVT), bilateral pulmonary embolism (PE), pulmonary artery aneurysm (PAA), and right‐sided intracardiac thrombus. Lower extremity venous thrombosis accounts for 70%–80% of all vascular involvement in HSS, as opposed to pulmonary artery involvement (PAI), which carries a prevalence of around 10% and tends to be bilateral, diffuse, and commonly localizing to the lower lobe arteries [6] (all of which apply to our patient). About two thirds of PAI in HSS owes to aneurysms, while one third is attributed to thrombosis [6].

Although BD can present with vascular complications, HSS is unique in its marked predilection for severe vascular involvement with limited systemic features [1, 2]. The presence of extensive pulmonary artery aneurysms and a large intracardiac thrombus without major mucocutaneous or ocular involvement makes HSS the more fitting diagnosis [4, 8, 9]. HSS is often rapidly progressive and life threatening due to pulmonary artery aneurysm rupture, which was a key concern in our case.

The EULAR 2018 guidelines recommend steroids and immunosuppressants as first‐line treatment for acute DVT, without a clear role for anticoagulation due to lack of evidence on its benefit in preventing relapses; however, there might be a potential role in refractory DVT [8]. They also recommend steroids and cyclophosphamide as the first‐line treatment for both PAA and thrombosis, in addition to anti‐TNF agents as an option for refractory cases [8]. Of note, aneurysm regression or resolution after immunosuppression is well documented in as short as 3 months [7, 10]. However, despite maintenance immunosuppression, about 20% of patients unfortunately experience relapse in 7 years, with 26% mortality [7]. Surgery should be reserved for life‐threatening cases since it carries a high mortality rate [6, 7, 8]. After starting patients on immunosuppression, interventional procedures like stenting or embolization can be performed on a case‐dependent basis if the patient has a high risk of bleeding or is symptomatic despite adequate medical therapy [6, 7].

PAA's pose a significant challenge to management, especially when they co‐exist with thrombosis, whether it be arterial, venous, or intracardiac; since they inform the decision of anti‐coagulation. All patients with HSS‐related thrombosis should be started on anticoagulation and should be screened for aneurysms to avoid catastrophic bleeding.

Right‐sided intracardiac thrombi are a distinctive feature of HSS. Some authors believe that any patient presenting with a right‐sided intracardiac thrombus should be suspected to have HSS and investigated accordingly, even if they do not have other classical features of the disease [9]. Intracardiac thrombi in HSS seem to be a surrogate for severe disease and are more likely to occur in young males at an average age of 28 [11], even earlier than other classical disease features. In a systematic review of 25 HSS patients with intracardiac thrombus, patients were treated with immunosuppression, anticoagulation, surgery, or a combination of the two or three modalities [9]. It was difficult to establish the superiority of one treatment approach over the other since mortality was secondary to pulmonary vasculature complications for the most part, regardless of the treatment received [9]. Some clinicians are in favor of anticoagulation in tandem with immunosuppression in cases of intracardiac thrombus based on evidence from retrospective studies, given the absence of PAA or relatively low bleeding risk [5, 9].

Our patient posed a challenge with respect to medical management due to concurrently having arterial and cardiac thrombosis in addition to pulmonary artery branch aneurysm and features of alveolar hemorrhage on imaging. Once the constellation of imaging features was deemed highly characteristic of HSS by the radiologist, rheumatologist, and cardiologist, he was appropriately started on pulse steroids, followed by maintenance dose and cyclophosphamide. The decision for anticoagulation was taken by the cardiologist due to the presence of a large right atrial thrombus: a treatment strategy well‐documented in the literature. Given the relatively small size of the pulmonary artery branch aneurysm (11 mm) and the fact that the patient was under observation while starting anticoagulation, the rheumatology team was on board. Classically, larger aneurysms of a size greater than 3 cm carry a higher risk for rupture and complications [7]. The decision to discharge the patient on a course of oral anticoagulation was cautiously taken by the cardiologist after learning that the aneurysm has regressed in size on repeat imaging owing to immunosuppression. Fortunately, the decision to forego thrombusaspiration—aa plan initially proposed by cardiology—was also made since the thrombus had decreased in size on repeat imaging after anticoagulation. While the cardiologist initially opted for life‐long anticoagulation, the rheumatologist rather argued in favor of a few months of anticoagulation followed by reassessment through echocardiography.

While there is no unified proposed duration of treatment due to the significant clinical heterogeneity of the disease, case reports on HSS with PAI have shown improved survival in patients treated with a tapered course of steroids followed by IV pulses of cyclophosphamide for at least 2 years, after which the latter could either be continued or replaced by maintenance azathioprine: an approach later recommended by the EULAR guidelines [8, 10]. Longer duration of treatment with the addition of endovascular interventions could be implemented in refractory cases.

4

This case report aims to remind the internist that concurrent venous and arterial insults, especially of a characteristic pattern like (a) bilateral, extensive pulmonary artery embolisms, (b) pulmonary artery aneurysms, and (c) lower extremity deep venous thromboses in addition to right‐sided intracardiac thrombi should immediately prompt entertaining a diagnosis of Hughes–Stovin Syndrome. Early involvement of the respective experts, including a rheumatologist and a cardiologist, helps to expedite the diagnosis and management, which is instrumental to morbidity and mortality outcomes. We also aim to alert the internist to screen all patients suspected to have HSS and expected to benefit from anticoagulation for aneurysms to avoid catastrophic bleeds. Moreover, all newly diagnosed HSS patients should also be screened for lower extremity venous thrombosis, which carries a high medical, social, and economic burden.

## Author Contributions

Conceptualization: Abdul Qadir and Amal Wael Abdellatif. Data curation: Abdul Qadir and Amal Wael Abdellatif. Writing – original draft: Abdul Qadir, Amal Wael Abdellatif, and Mamunul Islam. Writing – review and editing: Abdul Qadir, Amal Wael Abdellatif, Mamunul Islam, and Amir Waheed. All authors read and approved the final manuscript.

## Ethics Statement

The Medical Research Center at Hamad Medical Corporation in Qatar has granted approval for the publication of this case report.

## Consent

Written informed consent was obtained from the patient for publication of the details of their medical case and any accompanying images.

## Conflicts of Interest

The authors declare no conflicts of interest.

## Data Availability

The data that supports the finding of this case report are contained within the article. Additional information is available from the corresponding author upon reasonable request and with the approval of the relevant ethics committee.
